# Parental brain‐derived neurotrophic factor genotype, child prosociality, and their interaction as predictors of parents’ warmth

**DOI:** 10.1002/brb3.685

**Published:** 2017-04-04

**Authors:** Reut Avinun, Ariel Knafo‐Noam

**Affiliations:** ^1^ Department of PsychologyThe Hebrew University of JerusalemJerusalemIsrael

**Keywords:** fathers, gene–environment interaction, parenting, prosocial behavior, the brain‐derived neurotrophic factor gene, warmth

## Abstract

**Background:**

Parental warmth has been associated with various child behaviors, from effortful control to callous‐unemotional traits. Factors that have been shown to affect parental warmth include heritability and child behavior. However, there is limited knowledge about which specific genes are involved, how they interact with child behavior, how they affect differential parenting, and how they affect fathers. We examined what affects paternal and maternal warmth by focusing on the child's prosocial behavior and parents’ genotype, specifically a Valine to Methionine substitution at codon 66 in the brain‐derived neurotrophic factor (BDNF) gene.

**Methods:**

Data was available from a sample of 6.5 year‐old twins, consisting of 369 mothers and 663 children and 255 fathers and 458 children. Self‐reports were used to assess mothers’ and fathers’ warmth. Child prosociality was assessed with the other‐parent report and experimental assessments.

**Results:**

Mothers’ warmth was not affected by their BDNF genotype, neither as a main effect nor in an interaction with child prosociality. Fathers with the Met allele scored higher on warmth. Additionally, there was a significant interaction between fathers’ BDNF genotype and child prosociality. For fathers with the Met allele there was a positive association between warmth and child prosociality. Conversely, for fathers with the Val/Val genotype there was no association between warmth and child prosociality. Results were repeated longitudinally in a subsample with data on age 8–9 years. A direct within family analysis showed that fathers with the Met allele were more likely than Val/Val carriers to exhibit differential parenting toward twins who differed in their prosocial behavior. The same pattern of findings was found with mother‐rated and experimentally assessed prosociality.

**Conclusions:**

These results shed light on the genetic and environmental underpinnings of paternal behavior and differential parenting.

## 1 Introduction

Parental warmth has been associated with various child behaviors. High warmth has been associated with empathy (Zhou et al., 2002), prosocial behavior (Carlo, Mestre, Samper, Tur, & Armenta, 2011), and effortful control (Eisenberg et al., 2005), and low warmth has been associated with callous‐unemotional traits (Waller et al., 2014), low self‐regulation (Eiden, Colder, Edwards, & Leonard, 2009), and depressed mood and conduct problems in girls (Hipwell et al., 2008). Warmth has also been shown to moderate the association between harsh parenting and child externalizing problems (Deater‐Deckard, Ivy, & Petrill, 2006; McLoyd & Smith, 2002). Despite the importance of parental warmth, research on its etiology is relatively limited.

Various factors have been shown to affect parental warmth, such as heritability (Klahr & Burt, 2014); the parent's personality (Prinzie, Stams, Deković, Reijntjes, & Belsky, 2009); the child's behavior (Barnett, Gustafsson, Deng, Mills‐Koonce, & Cox, 2012); and socioeconomic status (SES), race, and a dangerous neighborhood (Pinderhughes, Nix, Foster, & Jones, 2001). However, there is limited knowledge about which specific genes the heritability estimate represents, how the factors that affect warmth interact, how they affect differential parenting, and how they affect fathers, as most of the research to date has been done on mothers. We focused on the genetic and child behavior factors and examined how children's prosocial behavior, which has been shown to elicit positive parenting (Barnett et al., 2012; Newton, Laible, Carlo, Steele, & McGinley, 2014; Padilla‐Walker, Carlo, Christensen, & Yorgason, 2012), may differentially affect paternal and maternal warmth according to the parent's genotype. In other words, we were interested in investigating whether children's prosocial behavior would be more likely to affect certain parents, who may be more sensitive to environmental cues as a result of their genetic makeup.

### 1.1 Child influences on parenting

A few decades ago it has been suggested that the parent‐child relationship should be viewed as reciprocal (Bell, 1968; Maccoby & Martin, 1983), as child influences on the surrounding environment, specifically parents, began to receive more attention. For example, longitudinal analyses suggested that at age 11, children's externalizing problems predicted less warm and responsive parenting, even after controlling for parenting at age 9 (Zhou et al., 2002). In a meta‐analysis of children‐as‐twins studies (Avinun & Knafo, 2014), it has been shown that 23% of the individual differences in parental behavior can be explained in terms of genetically influenced evocative child effects. While attention has somewhat shifted to include child influences in the study of socialization, most of the focus has been on negative child behaviors. Studies that did examine the bidirectionality of the association between positive parenting and prosocial behavior, have found evidence of child influences (Barnett et al., 2012; Carlo et al., 2011; Newton et al., 2014). Yet, depending on parental characteristics, some parents may be more or less susceptible to their child's behavior.

### 1.2 Genes as moderators of parental behavior

Research on interactions between specific genes and the environment has been gaining more attention in the last decade (Pluess & Belsky, 2013). Yet, to the best of our knowledge, research on how parental genes moderate the influence of the child behavior on parenting is rare and has never included a sample of fathers (Kaitz et al., 2010; Lee et al., 2008; Morgan, Hammen, & Lee, 2016). Lee et al. (2008), have found that mothers’ dopamine transporter genotype is associated with negative parenting. This association was significantly stronger among mothers whose children were highly disruptive. Kaitz et al. (2010), have found that variation in the dopamine receptor D4 moderates the association between infant fussiness and maternal sensitivity. And lastly, on a sample that mostly included mothers (85.6%), Morgan et al. (2016) have shown how the parents’ serotonin transporter genotype moderates the association between child noncompliance and parental negativity. We chose to focus on the brain‐derived neurotrophic factor (BDNF), which has been associated with both the serotonergic (Eaton, Staley, Globus, & Whittemore, 1995; Mamounas, Blue, Siuciak, & Altar, 1995) and dopaminergic systems (Guillin et al., 2001; Küppers & Beyer, 2001).

### 1.3 The brain‐derived neurotrophic factor gene

Brain‐derived neurotrophic factor is a member of the nerve‐growth‐factor family and it plays an important role in neuronal survival and development. In humans, the BDNF gene has a functional single‐nucleotide polymorphism, a Valine to Methionine substitution at codon 66 (Val66Met), which leads to impairments in intracellular trafficking and activity‐dependent secretion (Chen et al., 2004; Egan et al., 2003), and to various morphological changes in brain areas such as the hippocampus, prefrontal cortex, and the amygdala (reviewed in Notaras, Hill, & van den Buuse, 2015). The BDNF gene has been shown to interact with various environmental factors in affecting behavioral outcomes (Drury et al., 2012; Hayden et al., 2010; Willoughby, Mills‐Koonce, Propper, & Waschbusch, 2013). For example, homozygotes for the BDNF Met allele who spend more time with aggressive peers in middle childhood were found to be more likely to exhibit aggressive behavior in adolescence (Kretschmer, Vitaro, & Barker, 2013). In another study (Willoughby et al., 2013), it was shown that harsh–intrusive parenting is more strongly associated with oppositional defiant and callous‐unemotional behaviors for infants who are carriers of the Met allele. Because the BDNF gene appears to moderate sensitivity to environmental cues, it may be a potential moderator of the effects of child behavior on parenting.

### 1.4 Sex as a moderator of child and genetic effects

Sex has been shown to moderate the expression levels and effects of BDNF in various mammals (Advani, Koek, & Hensler, 2009; Monteggia et al., 2007) including humans (Choi, Bhang, & Ahn, 2011; Lommatzsch et al., 2005; Shalev et al., 2009). For example, in contrast to BDNF heterozygous (BDNF^+/−^) female mice, BDNF^+/−^ male mice exhibit increased immobility in the forced swim test after mild stress (Advani et al., 2009). In humans, the val66met polymorphism of the BDNF gene affects the onset of multiple sclerosis (MS) only in men and increases the risk of MS only in women (Mirowska‐Guzel, Mach, Gromadzka, Czlonkowski, & Czlonkowska, 2008). These differences may be explained by interactions between BDNF and sex hormones such as estradiol (Begliuomini et al., 2007; Sohrabji, Miranda, & Toran‐Allerand, 1995) and testosterone (Hill, Wu, Kwek, & van den Buuse, 2012; Verhovshek, Cai, Osborne, & Sengelaub, 2010). In rats, estrogen has been shown to regulate BDNF mRNA levels, possibly via an estrogen response element on the BDNF gene (Sohrabji et al., 1995). Castration of male rats reduced BDNF protein levels in motoneurons, an effect that was prevented with testosterone administration (Verhovshek et al., 2010). In women, for example, BDNF plasma concentrations have been found to change according to hormonal status (menstrual cycle, amenorrhea, and menopause; Begliuomini et al., 2007).

In addition, child influences have been shown to differ according to the parent's sex (Besnard et al., 2013; Marceau et al., 2015). For example, Karreman, van Tuijl, van Aken, and Deković (2008) have shown that mothers’ use of positive and negative control differed according to the child's level of effortful control, while fathers’ use of the same parenting dimensions did not. Elkins, McGue, and Iacono (1997) have found that father‐son relationships are more influenced by the sons’ genetically influenced behavior, than mother‐son relationships. They consequently postulated that because in certain social contexts less regular involvement is expected of fathers compared to mothers, the degree and nature of father‐child interactions are more influenced by the child's characteristics. Due to these observed sex differences we chose to examine mothers and fathers separately.

### 1.5 The current study

Considering the limited literature on the genetic underpinnings of maternal and paternal warmth and differential parenting, we set out to examine how mothers and fathers BDNF genotype may moderate the association between their warmth and children's prosocial behavior. Parents of 6.5‐year‐old twins reported on their parental warmth, and, to reduce method covariance, we used the other parent's report and experimental assessments to assess child prosociality. The children were also genotyped for the BDNF gene to rule out the possibility that genetic effects on parenting were affected by children's BDNF genotype. Since our data was cross‐sectional, we also used parental reports on warmth from a small subsample, that consisted of some of the same twins when they were 8–9 years old, to examine whether the interaction between the parent's BDNF genotype and child prosocial behavior can predict warmth at age 8–9, while controlling for warmth at age 6.5. Based on the literature reviewed above, we hypothesized that the parents’ BDNF genotype and child prosocial behavior would interact in predicting parental warmth, and that this interaction would differ according to the sex of the parent (we did not have a specific prediction regarding the direction of the sex effect).

## 2 Methods

### 2.1 Participants

Families in this study were participants in the Longitudinal Israeli Study of Twins (LIST), a study of social development, in which parents of all Hebrew‐ speaking families of twins born in Israel during 2004–2005 were invited to participate (Avinun & Knafo, 2013; Knafo, 2006). DNA was collected from mothers, fathers, and the twins. The protocol for the experiment was approved by the ethics committee of the Sarah Herzog Hospital, Jerusalem, and informed consent was obtained from all participating parents.

Thirteen families in which the twins were not genetically related to either parent were excluded from the study, as this may also affect parental behavior. The final sample of mothers with questionnaire assessments of child prosocial behavior consisted of 275 mothers and 510 children (51% males) with complete data on BDNF genotype (for both mother and children), mother reports on warmth, and SES, and father reports on children's prosocial behavior. The final sample of mothers with experimentally assessed child prosocial behavior consisted of 369 mothers and 663 children. The final sample of fathers with questionnaire assessments of child prosocial behavior consisted of 254 fathers and 468 children (49% males) with complete data on fathers’ and twins’ BDNF genotype, father reports on warmth, and mother reports on SES and each child's prosocial behavior. The final sample of fathers with experimentally assessed child prosocial behavior consisted of 255 fathers and 458 children. The study variables were not significantly associated with the presence of missing data in either the mother or the father samples.

### 2.2 Procedure

At age 6.5 (*M* = 6.62, *SD* = 0.25) the twins and their parents took part in lab or home assessments. Each twin entered into a separate room with an experimenter and underwent a series of social behavior tasks and situations as part of the LIST. Mothers and fathers also filled out questionnaires which included questions about parental behavior and child behavior.

### 2.3 Measures

#### 2.3.1 Parental warmth

Mothers and fathers reported regarding their expressions of warmth toward each twin by rating four items adapted from Robinson, Mandleco, Olsen, and Hart's (1995) Parenting Practises Questionnaire: “I am responsive to my child's feelings and needs,” “I provide comfort and understanding when my child is upset,” “I compliment my child when s/he behaves well,” and “I have warm and intimate times together with my child.” A 5‐point likert scale ranging from 1 = never to 5 = always, was used for each twin separately. A factor analysis confirmed that all items loaded on a single factor (loadings = 0.64–0.75 for mothers and 0.70–0.80 for fathers) explaining 50% of the variance in maternal warmth and 58% of the variance in paternal warmth. Cronbach's alpha was .66 for maternal warmth and .76 for paternal warmth. These factor scores were used for the analyses.

#### 2.3.2 Child prosocial behavior from parent reports

Mothers and fathers reported regarding each child's prosocial behavior by rating five items from the Strengths and Difficulties Questionnaire (Goodman, 1997): “Considerate of other people's feelings,” “Helpful if someone is hurt, upset, or feeling ill,” “Kind to younger children,” “Readily shares with other children” and “Often volunteers to help others (parents, teachers, other children).” Ratings were given on a 3‐point scale ranging from 0 (not true/rare) to 2 (very true/often). A factor analysis confirmed that all items loaded on a single factor (loadings = 0.55–0.73 for mother reports and 0.60–0.71 for father reports) explaining 42% of the variance in mother reports of prosocial behavior and 41% of the variance in father reports of prosocial behavior. The factor scores were used for the analyses. Cronbach's alpha was 0.63 for both mother and father reports of child prosociality. Father reports regarding prosocial behavior were used with the sample of mothers and mother reports were used with the sample of fathers.

#### 2.3.3 Experimentally assessed child prosocial behavior

Three situations relating to prosocial behavior were used in this study: (1) The experimenter “accidentally” drops a box of paper clips on the floor (Iannotti, 1985). She says, “Oops,” continues writing for 20 s, and then retrieves the paper clips for 10 s. The child who spontaneously helped during the initial 20 s received a score of 1. The child who helped when the experimenter was collecting the paper clips received a score of 0.5. (2) The experimenter gives the child a pack of candy similar to M&M's. She then takes a pack for herself while stating how much she likes it. After about 10 s she looks at her pack of treats and expresses disappointment for only having broken and melted candy. If the child offered to share his/her pack then s/he received a score of 1 (adapted from Yarrow et al., 1976). (3) The experimenter shows the child a box full of markers and says: “look, here is a box of markers, some of them work and some do not. There are children who do not have markers, and we would like to give them some. You can check on this page which markers work, and then we will give the good ones to children who do not have any. If you do not wish to do it, you can choose a page to color from this coloring book.” If the child chose to check the markers, then s/he received a score of 1. A total score of prosocial behavior was calculated by counting the behaviors in these three situations (as has been done with similar data from LIST; Knafo, Israel, & Ebstein, 2011). This score was used as an additional, but separate, assessment of child prosocial behavior.

#### 2.3.4 Child conduct problems from maternal reports

To address the specificity of parental reaction to prosocial behavior, we included conduct problems as an indicator of undesirable behavior. Mothers reported regarding each child's conduct problems by rating five items from the Strengths and Difficulties Questionnaire (Goodman, 1997): “Often has temper tantrums or hot tempers”; “Generally obedient, usually does what adults request”; “Often fights with other children or bullies them”; “Often lies or cheats”; and “Steals from home, school or elsewhere.” Ratings were given on a 3‐point scale ranging from 0 (not true/rare) to 2 (very true/often). A factor analysis showed that “Often lies or cheats” and “Steals from home, school or elsewhere” did not load significantly (loading < 0.11) on the main factor, and therefore a factor based on the three remaining items was created (loadings = 0.71–0.77) explaining 55% of the variance (Cronbach's alpha = .55). The factor scores were used for the analysis.

#### 2.3.5 DNA extraction and genotyping

Parents’ DNA was extracted from 20 ml of mouthwash samples using the Master Pure kit (Epicentre, Madison, WI, USA). Twins’ DNA (used as a control variable) was isolated from buccal epithelial cells using buccal swab brushes that were kept after collection in a sterile tube containing 15 ml of “Aquafresh” mouthwash. Genotyping was done as described by Shalev et al. (2009). The BDNF SNP was in Hardy–Weinberg equilibrium. Allele frequencies of the BDNF genotypes were as follows: Met/Met 3.3%–4.8%, Met/Val 31.8%–34.4%, Val/Val 62.3%–63.4% for children, mothers, and fathers. Met carriers were examined versus the Val/Val carriers. The Met carriers were grouped and coded as 0, while the Val/Val carriers were coded as 1.

#### 2.3.6 Socioeconomic status

Socioeconomic status was calculated based on mother reports of number of rooms/number of residents ratio, income below or above average household income, and mother's years of education. These were standardized and averaged to create an SES score.

#### 2.3.7 Statistical analysis

Mendelian inheritance and Hardy–Weinberg equilibrium were checked and verified with PEDSTATS version 0.6.12. (Wigginton & Abecasis, 2005). Multilevel analysis with families as clusters was done in Mplus version 7 (Muthén & Muthén, 2007), under the default option of maximum likelihood estimation with robust standard errors, which is robust to non‐normality and allows for multilevel analyses based on unbalanced groups. The multilevel analysis allowed using both twins in the same analysis. BDNF genotype and child prosocial behavior were grand mean centered before computing the interaction terms to avoid multicollinearity. Child BDNF genotype, child sex, and SES were entered into the models as control variables. Thus, in level 1 (variables that differ within the family) of the multilevel analysis, child sex, child BDNF genotype, child prosocial behavior, and the interaction term were entered, and in level 2 (variables that differ between families), the parent's BDNF genotype and SES were entered. Analyses were done separately for parent reports and experimental assessments of children's prosocial behavior, as we were interested in examining whether the results would replicate with two different measures of prosociality.

As an additional analysis we used the full information maximum likelihood (FIML) method to handle the missing data in the father sample. All model variables were allowed to have missing values except the BDNF genotype (children and fathers) and maternal warmth. The latter was used as a condition in order to add valuable information to the model.

The significant moderation effects were followed up using a web utility for simple slopes and regions of significance (Preacher, Curran, & Bauer, 2006). To examine the direction of the significant moderation effects, we conducted a longitudinal analysis on a small subsample. Lastly, to directly test for within family effects and compare the behavior of the same father toward two twins that differed in their levels of prosocial behavior, paired samples *t* tests were conducted in SPSS (RRID:SCR_002865) version 19 for windows.

## 3 Results

### 3.1 Descriptive statistics and correlations

Descriptive statistics are presented in Table 1. Mothers’ and fathers’ mean warmth and variance were compared in a sample of 643 twins and 347 families for whom data was available on both parents. To take into account the dependency of mothers and fathers and the dependency of the twins Pitman's *t* test was used with the variances estimated in Mplus with type=complex. Mothers (*M *=* *4.54, *SD* = 0.44) and fathers (*M *=* *4.44, *SD* = 0.50) significantly differed in their average levels of warmth and in their variances, *t*(641) = 4.42, *p *<* *.001; *t*(641) = 3.42, *p *<* *.001, respectively. Fathers reported lower levels of warmth and were characterized by a higher variability. Experimentally assessed estimates of prosocial behavior correlated with mother‐reported, *r*(875) = .13, *p *<* *.001 and father‐reported prosocial behavior, *r*(647) = .13, *p *<* *.001. The correlation between mother and father reports was *r*(675) = .41, *p *<* *.001 (Table 1).

**Table 1:** Correlations (Ns), means and standard deviations for warmth and child prosocial behavior

	1	2	3	4	5
1. Maternal warmth	—				
2. Paternal warmth	.253 (643)**	—			
3. Mother‐reported child prosocial behavior	.156 (886)**	.121 (660)**	—		
4. Father‐reported child prosocial behavior	.118 (658)**	.282 (701)**	.408 (675)**	—	
5. Experimentally assessed child prosocial behavior	.027 (849)	.037 (686)	.129 (875)**	.121 (704)**	—
*SD*	0.43	0.50	0.40	0.40	0.84
Means (*N*)	4.53 (935)	4.45 (691)	1.54 (957)	1.51 (720)	1.75 (901)

For clarity, the non‐standardized scores are presented. The Ns differ because each statistic is based on the entire available data.

**p <* *.01.

### 3.2 Hierarchical linear modeling results for the mother sample

In the samples of mothers (a sample in which child prosociality was based on father reports and a sample in which it was based on experimental assessments), mothers’ and children's BDNF genotypes, child prosocial behavior, SES, child sex, and the interaction between mothers’ BDNF genotype and child prosocial behavior, were not significant predictors of warmth, as can be seen in Table 2.

**Table 2:** Warmth as an outcome in mother and father samples

	Mothers (observed)	Fathers (observed)	Fathers (observed)^a^	Mothers (questionnaires)	Fathers (questionnaires)	Fathers (questionnaires)^a^
	*b* (*SE*)	*b* (*SE*)	*b* (*SE*)	*b* (*SE*)	*b* (*SE*)	*b* (*SE*)
Within						
Parental warmth age 6.5 (within variance)			−.13 (.13)			−.07 (.11)
Child sex	−.00 (.09)	−.01 (.10)	−.27* (.13)	.02 (.11)	−.01 (.10)	−.18 (.13)
Child BDNF	−.01 (.09)	.03 (.11)	−.00 (.13)	−.03 (.10)	−.01 (.10)	.03 (.12)
Child prosociality	.04 (.04)	.05 (.04)	.17** (.06)	.04 (.04)	.08** (.03)	−.02 (.05)
Parent BDNF × Child prosociality	−.01 (.09)	−.18* (.08)	−.27* (.13)	−.03 (.08)	−.17**(.06)	−.06 (.12)
Between						
Parental warmth age 6.5 (between variance)			.54** (.14)			.55** (.14)
Parent BDNF	.12 (.11)	−.27* (.13)	−.17 (.15)	.12 (.14)	−.26* (.13)	−.12 (.15)
SES	.02 (.06)	.06 (.08)	.12 (.10)	.04 (.07)	.06 (.07)	.13 (.11)

Showing the results for both parent‐reported (questionnaires) and experimentally assessed (observed) child prosocial behavior. BDNF, brain‐derived neurotrophic factor.

a Predicting change in paternal warmth from age 6.5 to age 8–9.

*p <* *.05; **p <* *.01.

### 3.3 Hierarchical linear modeling results for the father sample

#### 3.3.1 Child‐level effects

The effect of child sex on paternal warmth was not significant, nor was the effect of the child's BDNF genotype (Table 2). Higher levels of paternal warmth were associated with higher levels of child prosociality when it was rated by mothers (*b *=* *.09, *SE* = .03, *p *<* *.01), but not when it was experimentally assessed (*b *=* *.05, *SE* = .04, ns).

#### 3.3.2 Father‐level effects

Fathers’ BDNF genotype had a main effect on warmth, so that fathers who were Met carriers were characterized by higher levels of warmth than Val/Val carriers (mother reports: *b *=* *−.26, *SE* = .13, *p *<* *.05; experimental assessments: *b *=* *−.27, *SE* = .13, *p *<* *.05). The effect of SES on paternal warmth was not significant.

#### 3.3.3 Interaction effect

The effect of an interaction between child prosociality and fathers’ BDNF genotype on paternal warmth was significant both when child prosociality was rated by mothers (*b *=* *−.16, *SE* = .06, *p *<* *.01) and when it was experimentally assessed (*b *=* *−.18, *SE* = .08, *p *<* *.05).

**Figure 1:**
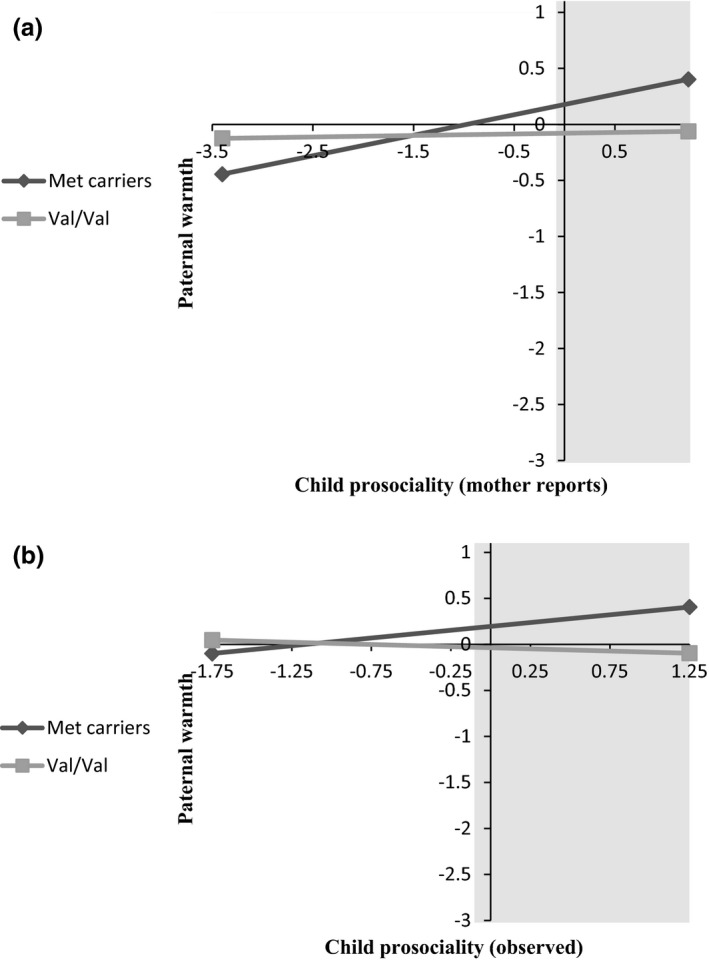
Interaction between fathers’ brain‐derived neurotrophic factor genotype and child prosociality in predicting paternal warmth *The grey area marks the region of significance

Follow‐up simple slopes analysis on the sample of fathers with child prosociality as reported by mothers, revealed that the simple slope of Met carriers (*b *=* *.18) was significant (*Z *=* *3.82, *p *=* *.0001), indicating that they showed higher levels of warmth when their child was more prosocial. The slope of the Val/Val carriers (*b *=* *.01) was not significant (*Z *=* *0.36, ns), indicating that their level of warmth did not depend on child prosocial behavior. Region of significance analysis (Preacher et al., 2006) indicated that above −.04 in child prosociality as reported by mothers (slightly below the −.02 mean), the two regression lines significantly differed (Figure 1a). Similar results were found for the father sample with experimentally assessed prosociality (Figure 1b). The simple slope of fathers who were Met carriers (*b *=* *.17) was significant (*Z *=* *3.33, *p *=* *.0009), while the slope of the Val/Val carriers (*b *=* *−.02) was not significant (*Z *=* *−0.30, ns). Furthermore, region of significance analysis revealed that from slightly below the mean (−0.104, when the mean was 0.01 and the *SD* was 0.84) of experimentally assessed child prosociality onward, Met carriers were significantly higher than Val/Val carriers in levels of warmth.

#### 3.3.4 Handling missing data

Because many fathers were missing from our sample of families, we also tested the models while applying the FIML method. For 629 children and 340 fathers the results of the two models (experimentally assessed and mother rated prosociality) were very similar to the original models with no missing data. In the experimentally assessed prosociality model the main effect of the BDNF polymorphism was significant (*b *=* *−.26, *SE* = .12, *p *<* *.05) and the interaction was close to significance (*b *=* *−.13, *SE* = .07, *p *=* *.057). In the mother rated prosociality model the main effects of prosociality and BDNF genotype were significant (*b *=* *.07, *SE* = .03, *p *<* *.01; *b *=* *.27, *SE* = .05, *p *<* *.05) and the interaction was significant (*b *=* *−.13, *SE* = .05, *p *<* *.05).

#### 3.3.5 Testing whether the interaction effect is specific to prosocial behavior

We wanted to examine whether the effect of the interaction between fathers’ BDNF genotype and child prosocial behavior on paternal warmth is specific to the positive influence of child prosocial behavior. Consequently, we added an interaction between fathers’ BDNF genotype and conduct problems as reported by mothers to the original models. The interaction between the fathers’ BDNF genotype and child prosocial behavior in predicting paternal warmth remained significant (mother reports of prosocial behavior: *b *=* *−.14; *SE* = .06; *p *<* *.05; experimentally assessed prosocial behavior: *b *=* *−.19; *SE* = .08; *p *<* *.05), while the interaction between fathers’ BDNF genotype and conduct problems was not a significant predictor of paternal warmth (mother reports model: *b *=* *.02; *SE* = .07; ns; experimental assessment model: *b *=* *.04; *SE* = .07; ns).

#### 3.3.6 Probing the direction of the effect

Because our data is cross‐sectional, we also examined whether the reverse interaction, from parent to child, is significant. In other words, we tested the interaction between the child's BDNF genotype and fathers’ warmth in predicting child prosocial behavior as rated by mothers or as experimentally assessed. This parent to child interaction was not significant in either case (mother reports: *b *=* *.02, *SE* = .08, ns; experimental assessments: *b *=* *.09, *SE* = .08, ns).

We also capitalized on the longitudinal design of the LIST sample (Avinun & Knafo, 2013) and used the data we had available on the same families from age 8–9 to test for a longitudinal effect. Of those families who participated at age 6.5, full data on paternal warmth, assessed by the same questionnaire from age 6.5, was available for 102 fathers and their 183 children (176 when using experimentally assessed prosocial behavior data) when the twins were 8–9 (*M *=* *8.88 years, *SD* = 0.38). Paternal warmth at age 6.5 was correlated with paternal warmth at age 8–9 (*r *=* *0.51, *p *<* *.001). As shown in Table 2, we tested whether an interaction between prosocial behavior at age 6.5 and fathers’ BDNF genotype could predict paternal warmth at age 8–9, while controlling for paternal warmth at age 6.5 and for the covariates from the original model (i.e., child's sex, child's BDNF genotype, and SES). The interaction between fathers’ BDNF genotype and child prosocial behavior at age 6.5, did not predict paternal warmth at age 8–9 (*b *=* *−.06, *SE* = .12, ns) when prosocial behavior was rated by mothers, but did significantly predict paternal warmth at age 8–9 when it was experimentally assessed (*b *=* *−.27, *SE* = .13, *p *<* *.05). Thus this longitudinal analysis replicated and strengthened the cross‐sectional finding, by showing that the interaction between fathers’ BDNF genotype and experimentally assessed child prosocial behavior predicted paternal warmth at age 8–9, even when warmth at age 6.5 was used as a control variable.

#### 3.3.7 A direct test of within family differences

We utilized the sample of twins to conduct within family comparisons and directly test whether a father who is a Met carrier would be more likely than a father who is a Val/Val carrier, to be differentially warm toward his twins due to a difference in their level of prosocial behavior. In other words, we only included twin pairs that differed in their prosocial scores (183 twin pairs with mother reports on prosocial behavior and 126 twin pairs with experimentally assessed prosocial behavior) and tested whether the warmth they received from their fathers differed as a function of the fathers’ BDNF genotype. We created two groups: Group 1 consisted of the children who showed higher levels of prosocial behavior within a twin pair and Group 2 consisted of the children who showed lower levels of prosocial behavior within a twin pair. Paired samples *t* tests of paternal warmth based on fathers’ BDNF genotype revealed the expected results. There was no significant difference in warmth between Group 1 and Group 2 for fathers with the Val/Val genotype (mother reports: mean difference = −0.004, *SD* = 0.65, *t*(109) = −.07, ns; experimental assessments: mean difference = 0.006, *SD* = 0.85, *t*(74) = 0.06, ns). In contrast, Met carriers did show more warmth toward the more prosocial child (mother reports: mean difference = 0.21, *SD* = 0.57, *t*(72) = 3.09, *p *<* *.01; experimental assessments: mean difference = 0.19, *SD* = 0.53, *t*(50) = 2.60, *p *<* *.05). This analysis further strengthens our initial findings and also lends support to a child‐driven effect, by showing that twins from the same family, who differ in their levels of prosocial behavior, only differ in the levels of warmth they receive from their fathers, when their fathers are Met carriers.

## 4 Discussion

Our results add to the limited research on how parental genes affect parenting and how they moderate the association between parenting and child behavior. To our knowledge, the current study is the first to show an association between a specific gene and paternal behavior. Specifically, we found that fathers who are carriers of the BDNF Met allele are characterized by higher levels of warmth compared to fathers who are carriers of the Val/Val genotype. Additionally, we found an interaction between the fathers’ BDNF genotype and child prosocial behavior. Fathers carrying the Met allele showed higher levels of warmth toward more prosocial children, than toward less prosocial children. In contrast, there was no association between fathers’ warmth and child prosociality, when the fathers were homozygotes for the Val allele. These results were similar for both mother reports and experimental assessments of children's prosocial behavior. In addition, the results were repeated with a subsample on which data from age 8–9 was available, thus showing that child prosociality can predict paternal warmth in interaction with fathers’ BDNF genotype. Notably, the interaction between fathers’ BDNF genotype and child prosocial behavior remained significant after controlling for an interaction between fathers’ BDNF genotype and child conduct problems. This supports the interpretation that the positive behavior of the child has a unique influence on paternal warmth. Conversely, mothers’ warmth was not affected by their BDNF genotype, neither as a main effect nor in an interaction with child prosociality.

The interaction effect between fathers’ BDNF genotype and child prosociality in predicting paternal warmth, was evident both when child prosociality was estimated by mother reports and when it was experimentally assessed. Notably, the correlation between the mother reports and the experimental assessments was relatively low, which implies that two different aspects of prosocial behavior may have been estimated. It has been previously argued that prosocial behavior can be divided into three subtypes that stem from distinct social cognitive abilities: helping, sharing, and comforting (Dunfield, 2014). Additionally, compliant and self‐initiated prosocial acts have also been shown to be distinct aspects of prosocial behavior (Eisenberg, Cameron, & Tryon, 1984). Consequently, low correlations between different estimates of prosocial behavior are not surprising. While the two estimates of prosocial behavior that were used in the current study significantly, and separately, interacted with fathers’ BDNF genotype in predicting paternal warmth, it may be of interest to investigate in future research whether certain subtypes of prosocial behavior elicit more warmth in fathers than others.

The main effect of BDNF on paternal warmth, and its interaction with child prosociality, may be related to the association between BDNF and neuroticism. A meta‐analysis has shown that homozygotes for the BDNF Val allele are characterized by higher levels of neuroticism (Frustaci, Pozzi, Gianfagna, Manzoli, & Boccia, 2008), and higher levels of neuroticism have been linked to lower levels of warmth (Prinzie et al., 2009). Additionally, it has been found that less neurotic fathers change their use of positive control according to their child's level of effortful control, while fathers with relatively high levels of neuroticism do not (Karreman et al., 2008). It is possible that fathers who are homozygous for the Val allele are more neurotic, and are thus more likely to have a negative perspective and be less sensitive to the positive influence of their child. Notably however, the mentioned study did not find the same interaction effect (fathers’ neuroticism × child effortful control) on paternal warmth. Understanding which father characteristic mediates the observed effect of the BDNF polymorphism is an avenue for future research.

In addition, considering the involvement of BDNF in synaptic plasticity (Edelmann, Leßmann, & Brigadski, 2013), it is possible that variation in the BDNF gene affects a biological sensitivity to environmental cues more generally. Even though we demonstrated that an interaction between fathers’ BDNF genotype and child conduct problems did not predict paternal warmth, it is possible that fathers with the Met allele will react by adapting a different parental behavior, such as harsh discipline or love withdrawal, in accordance with the child's level of conduct problems. The exact mechanism that underlies the moderating effect of BDNF on paternal warmth, needs to be examined in future studies.

In the current study, the BDNF gene was found to be related to paternal, but not maternal, warmth. Various studies in mice (Advani et al., 2009; Ren‐Patterson et al., 2006) and in humans (Shalev et al., 2009; Verhagen et al., 2010) have shown a sex difference in the effect of BDNF. This difference may be related to the interactions between BDNF and sex hormones such as estradiol (Begliuomini et al., 2007; Sohrabji et al., 1995) and testosterone (Hill et al., 2012; Verhovshek et al., 2010). Another explanation for the sex difference in the interaction effect might lie in the lower variability found for maternal warmth compared to paternal warmth. Mothers may feel more socially obligated to rate themselves as warm or to behave warmly than fathers, and may therefore be, or appear to be, less influenced by their child. Further research is needed to clarify these sex differences.

The current findings implicate the BDNF genotype of fathers in differential parenting, and indicate that in response to different environmental cues from the child, fathers who carry the BDNF Met allele are more likely to express a different level of warmth. Differential parenting has been associated with negative child outcomes such as depressive symptoms (Shanahan, McHale, Crouter, & Osgood, 2008), oppositional behavior (Meunier, Bisceglia, & Jenkins, 2012), and antisocial behavior (Caspi et al., 2004). The current findings mark the parents’ genetic makeup as a potential candidate for future research on the etiology of differential parenting.

The strengths of the study include the use of both father and mother data and the use of different modes of assessment (genotype, parent‐reports, and other parent‐report/experimental assessments) which reduces method covariance. In addition, we replicated the main findings using both parent ratings and experimentally assessed child prosociality. One limitation is the cross‐sectional nature of the data, which means that the directionality of the effect should be treated with caution until the results are replicated with a large longitudinal sample. Notably however, a parent to child interaction between the child's BDNF genotype and warmth in predicting child prosocial behavior was not significant, a longitudinal analysis on a small subsample that included observed prosocial behavior from age 6.5 and warmth from age 8–9, while controlling for warmth from age 6.5, replicated the finding, and in an analysis that was based on twin differences in prosocial behavior and on the fathers’ genotype, fathers’ warmth only differed when the father was a Met carrier, thus providing support for a child to parent interpretation of causality. Another limitation is the reliance on self‐reports to assess parenting. Our results need to be replicated with a different measure of parental behavior. Even though observations have their own limitations (e.g., observer bias, relatively narrow spectrum of behaviors, parents awareness of being viewed/filmed), they may contribute to the understanding of our findings. In addition, it will be of interest to investigate whether other aspects of positive parenting, such as responsiveness and involvement, may change in accordance with child prosociality. Lastly, our results should be viewed as tentative until replicated with other samples.

Most studies to date have focused on maternal behavior and on negative child behaviors. The current study shows that as a function of their genotype, certain fathers may be more likely to change their warmth according to their children's behavior. The found effect is relatively modest, which is expected in a candidate gene study of a complex social behavior that is affected by multiple genes and environments. By making the first step in uncovering the genetic underpinnings of paternal warmth, we hope to open the door to future studies that will investigate additional genetic markers and the mechanisms through which they exert their effects. Gaining a deeper understanding of the genes and gene‐environment interactions that affect paternal warmth can advance parental and child‐parent treatments by shedding light on the involved brain circuits and by helping to differentiate between parents who are more reactive to their children's behavior and parents who are less reactive. Furthermore, our findings support the notion that the influences of positive child behaviors need to be considered in order to develop a full understanding of parenting and family processes, and suggest that fathers’ and mothers’ behavior may differ both genetically and in terms of child influences.

## Conflict of Interest

None declared.
